# Clinical outcomes and predictors of response to photodynamic therapy in symptomatic circumscribed choroidal hemangioma: A retrospective case series

**DOI:** 10.1371/journal.pone.0197088

**Published:** 2018-05-31

**Authors:** Yeen-Fey Ho, Anne Chao, Kuan-Jen Chen, An-Ning Chao, Nan-Kai Wang, Laura Liu, Yen-Po Chen, Yih-Shiou Hwang, Wei-Chi Wu, Chi-Chun Lai, Tun-Lu Chen

**Affiliations:** 1 Department of Ophthalmology, Chang Gung Memorial Hospital Linkou Medical Center, Taoyuan, Taiwan; 2 College of Medicine, Chang Gung University, Taoyuan, Taiwan; 3 Department of Ophthalmology, Yeezen General Hospital, Taoyuan, Taiwan; 4 Department of Anesthesiology, National Taiwan University Hospital, Taipei, Taiwan; Harvard Medical School, UNITED STATES

## Abstract

**Background:**

To investigate the treatment outcomes and predictors of response to photodynamic therapy (PDT) in patients with symptomatic circumscribed hemangioma (CCH).

**Methods:**

This retrospective case series examined 20 patients with symptomatic CCH (10 submacular CCHs and10 juxtapapillary CCHs) who underwent standard PDT (wavelength: 662 nm; light dose: 50J/cm^2^; exposure time: 83 sec) with verteporfin (6mg/m^2^), either as monotherapy (n = 9) or in association with other treatments (n = 11), of which 7 received intravitreal injections (IVI) of anti-vascular endothelial growth factor (anti-VEGF). A post-PDT improvement of at least two lines in best-corrected visual acuity (BCVA) was the primary outcome measure. Predictors of response were investigated with binary logistic regression analysis.

**Results:**

Seventeen (85%) patients received one PDT session, and three patients (15%) underwent PDT at least twice. Ten patients (50%) achieved the primary outcome of a post-PDT BCVA improvement of at least two lines. Macular atrophy and recalcitrant cystoid macular edema in 2 patients. Binary logistic regression analysis revealed that younger age (< 50 years) (*P* = 0.033), pre-PDT BCVA of ≧20/200 (*P* = 0.013), exudative retinal detachment resolved within one month after PDT (*P* = 0.007), and a thinner post-PDT tumor thickness (*P* = 0.015) were associated with the achievement of a post-PDT BCVA improvement. Additional treatments to PDT including IVI anti-VEGF did not appear to improve visual and anatomical outcomes.

**Conclusions:**

Symptomatic CCHs respond generally well to PDT. Patients with younger age (< 50 years), pretreatment BCVA≥ 20/200, and thinner foveal edema are most likely to benefit from this approach.

## Introduction

A circumscribed choroidal hemangioma (CCH) is an uncommon, benign choroidal tumor usually located at the posterior pole of the affected eye. Choroidal hemangiomas (CCHs) present as round to oval, reddish-orange choroidal tumors of varying size. They can be asymptomatic, being diagnosed during routine eye examinations or may cause decreased visual acuity because of exudative retinal detachment or cystoid macular edema (CME) [1,2,3]. Different therapeutic approaches including laser photocoagulation, transpupillary thermal therapy (TTT), radiation plaque therapy, external beam radiation, and proton beam radiation have been proposed to treat extramacular CCHs [1,2,4]. However, the use of either laser photocoagulation or TTT is limited by the risk of irreversible foveal damage associated with these methods. Moreover, post-radiotherapy radiation retinopathy is a significant concern [2,4,5]. Owing to a low likelihood of treatment-related visual loss [6,7,8,9,10,11,12], photodynamic therapy (PDT) with verteporfin is currently considered as the first-line treatment modality for patients with CCHs with macular involvement [2].

However, the variables associated with positive post-PDT visual improvements which may be helpful to select ideal candidates for this treatment approach have not been yet identified. Interest in the different protocols of PDT or with other treatment approaches for CCHs is increasing. Some studies have demonstrated that the intravitreal injection (IVI) of anti-vascular endothelial growth factor (anti-VEGF), such as bevacizumab or ranibizumab, may be effective in treating patients with CCH when applied alone or in combination with PDT, particularly in the presence of subretinal fluid (SRF) accumulation [13,14,15,16]. Herein, we report the visual and anatomical outcomes of 20 patients with symptomatic CCH who were treated with PDT, either as monotherapy or in combination with other treatments. We also sought to identify the main predictors of response to PDT in CCH patients, with the primary goal of identifying candidates who are most likely to benefit from this approach.

## Patients and methods

This study was a single-center retrospective case series. The protocol followed the tenets of the Declaration of Helsinki and was approved by the Institutional Research Ethics Board of the Chang Gung Memorial Hospital (CGMH, 104-A191B, 105-6752C), Linkou, Taiwan. Because of the retrospective nature of the study, the need for informed consent was waived.

### Study patients

We retrospectively reviewed the records of all patients with symptomatic CCH who were diagnosed and treated at the Department of Ophthalmology of the CGMH between January 1, 2006 and December 31, 2015. The diagnosis of CCH was based on the results of ophthalmoscopy, optical coherence tomography (OCT, Heidelberg Spectralis), fluorescein angiography (FA), indocyanine green angiography (ICGA), and ultrasonography (Nidek, US4000). On Fluorescein angiography (FA), choroidal hemangiomas show hyperfluorescence at early and late phases, subretinal fluid is visualized with more enhanced hyperfluorescence. The size and borders of CCHs can be delineated more clearly through ICGA than through FA. On ICGA, choroidal hemangiomas usually show hyperfluorescence at 1 minute, and the dye is washed out at 20 minutes, the tumor appears hypofluorescent relative to the surrounding choroid. CCHs with a thickness of <3 mm and a diameter <5 mm were considered as small tumors in accordance with the classification of the Collaborative Ocular Melanoma Study (COMS) [17]. Data on the following variables were collected in all participants: sex, age, tumor laterality, symptom duration (in months), decimal best-corrected visual acuity (BCVA), tumor location, maximum baseline tumor diameter, tumor thickness, foveal center thickness (FCT), number of PDT sessions, additional treatment modalities, and complications.

### Photodynamic therapy and additional treatment modalities

All patients underwent standard PDT (wavelength: 689 nm; light dose: 50 J/cm^2^; exposure time: 83 sec per spot. The Coherent Lumenis Opal PDT laser, Lumenis, Inc, Santa Clara, California, USA), with verteporfin (6 mg/m^2^ body surface; Visudyne, Norvatis Ophthalmics, Hettlingen, Switzerland) was used for treatment[8,9,10,11,12,18]. Multiple minimal overlapping spots were used to treat large CCHs. Depending on the physician’s discretion, and tumor response to previous treatments, including focal laser photocoagulation, IVIs of anti-VEGF, selected patients (n = 11) had received additional treatments to PDT, including (1) IVIs of different anti-VEGF drugs, or (2) radiation therapy. IVIs of bevacizumab (1.25 mg) were administered 1 week before PDT as a combination therapy to seven patients. (3) Retreatment with PDT alone or combined with IVI anti-VEGF was suggested 2 months after PDT if the tumors did not respond adequately to PDT, such as when persistent SRF, regrowth of tumors, or recurrence of SRF was observed.

### Outcome measures

The primary outcome measure was a positive response to PDT, defined as (1) a post-PDT improvement of at least two lines in BCVA scores compared with baseline (measured using the Snellen chart) is considered improved visual acuity as described in previous study and (2) a BCVA ≧0.3(20/60) at the last follow-up visit [19]]. The Snellen chart is used to measure visual acuity. Normal vision is presented by a score of 20/20 (1.0), which indicates most individuals can see the letter at a distance of 20 feet. If a 10 times magnification is required, the corresponding visual acuity is 1/10(20/200). The LogMAR notation is widely used in scientific publication to graphically depict visual acuity values for analysis or calculation of average values. A Snellen score of 20/20, indicates that the corresponding visual acuity can resolve a visual angle of 1 minute, corresponding to a LogMAR value of 0. Similarly, a Snellen score of 20/40, indicates that the visual acuity can resolve a visual angle of 2 minutes, corresponding to a Log MAR of 0.3.

Secondary outcome measures included (1) Pretreatment and post treatment FCT was measured by OCT using the software in the OCT machine, (2) Pretreatment and post treatment tumor thickness was measured by ultrasonography manually by AN Chao (3) the occurrence of local and systemic complications. Transient post-procedural exudative retinal detachment was defined as a post-PDT increase in SRF accumulation that was resolved within 1 month.

### Statistical analysis

Continuous data are presented as median values with range and were analyzed using the nonparametric Mann-Whitney *U* test. The Wilcoxon signed-rank test was used for paired comparisons. Categorical variables were presented as counts and were compared using the Chi-squared test. The association between the study variables and the primary outcome measure was evaluated using binary logistic regression. Results are presented as odds ratios (ORs) with their 95% confidence intervals (CIs). SPSS statistical software, version 22.0 (IBM, Armonk, NY, USA) was used for all calculations. Two-tailed *P* values <0.05 were considered statistically significant.

## Results

### General characteristics of the study patients

We reviewed the data of 20 patients (16 male and 4 female patients). Their median age was 46.0 years (range: 29−71 years). The median symptom duration at presentation was 2.0 months (range: 0.5−39 months). CCHs were located at macular area in 10 (50%) patients and in the juxtapapillary region in the remaining 10 (50%) patients. All of the patients presented with peritumor exudative retinal detachment with macular involvement. The median maximum tumor diameter at baseline was 7.25 mm (range: 5.0−15.0 mm). The details of the characteristics of the patients are listed in **Table 1**.

**Table 1:** Clinical characteristics of patients with circumscribed choroidal hemangioma and serous macular detachment in whom received photodynamic therapy with verteporfin.

	Sex	Age (years)	Laterality	Symptom duration (months)	Decimal BCVA		Tumor location	Initial tumor basal diameter (mm)	Tumor thickness (mm)		FCT (μm)		Sessions of PDT	Additional treatments	Complications
					Initial	Last visit		Maximal dimension	Initial	Post-PDT	Initial	Last visit			
1	M	29	OD	6.0	0.50	0.80	JP	7.0	3.4	1.2	450	206	2	-	Tumor regrowth
2	M	43	OS	4.0	0.10	0.05	JP	9.0	5.3	4.0	300	277	1	Pre-PDT FRP	
3	M	71	OS	6.0	0.03	0.05	JP	10.0	5.0	2.0	300	210	1	-	Macular atrophy
4^*a*^	M	41	OS	0.5	0.30	0.90	JP	7.5	3.6	0.9	487	233	1	-	-
5	F	52	OD	1.0	0.02	0.05	JP	10.0	4.9	3.9	327	823	1	Combined IVI-B	Recalcitrant SRF
6	F	58	OS	12.0	0.02	0.03	MA	15.0	7.2	6.5	837	982	5	Pre-PDT FRP; combined IVI-B, R	Recalcitrant SRF
7	F	46	OD	0.5	0.30	0.70	MA	7.0	3.0	2.4	360	174	1	-	-
8	M	56	OD	39.0	0.05	0.05	MA	6.0	3.0	4.2	802	945	1	Combined IVI-B	Recalcitrant SRF
9^*a*^	M	35	OD	4.0	0.40	0.70	MA	6.0	2.5	1.0	369	175	1	-	-
10	M	43	OS	2.0	0.05	0.05	JP	10.0	3.0	3.0	314	550	1	Pre-PDT FRP	
11	M	63	OD	1.0	0.05	0.05	MA	9.0	6.3	3.6	497	273	3	combined IVI-B, post- PDT RT	Macular atrophy
12	M	59	OD	26	0.03	0.05	MA	5.0	3.3	3.0	549	250	1		Macular atrophy
13	M	60	OS	1.0	0.40	0.30	MA	5.5	4.0	4.0	590	340	1	Combined IVI-R	-
14	M	46	OS	0.5	0.05	1.00	JP	5.0	5.0	2.0	823	255	1	-	-
15	M	39	OD	2.0	0.05	0.05	MA	7.0	6.0	5.0	626	309	1	-	Macular atrophy
16	M	40	OS	1.0	0.10	0.30	MA	5.5	4.6	3.6	592	269	1	Pre-PDT FRP	-
17	M	54	OD	0.5	0.06	1.00	JP	5.0	5.1	2.0	852	247	1	-	-
18	M	39	OD	26.0	0.10	0.30	JP	9.0	2.5	2.0	497	421	1	Combined IVI-B	-
19^*a*^	F	51	OS	12.0	0.20	0.40	JP	13.0	3.5	1.5	341	212	1	Pre-PDT FRP	-
20	M	38	OD	2.0	0.20	0.70	MA	10.5	4.0	3.1	327	256	1	Pre-PDT FRP; combined IVI-B	-

^*a*^These patients (n = 3) showed a transiently increased subretinal fluid after PDT, which improved one month thereafter.

Abbreviations: BCVA = best corrected visual acuity; FCT = foveal central thickness; PDT = photodynamic therapy; M = male; F = female; OD = right eye; OS = left eye; JP = juxtapapillary; MA = macular area; FRP = focal retinal laser photocoagulation; IVI = intravitreal injection; B = bevacizumab; R = ranibizumab; RT = radiation therapy; ERD = exudative retinal detachment.

### Treatment modalities

**Fig 1:**
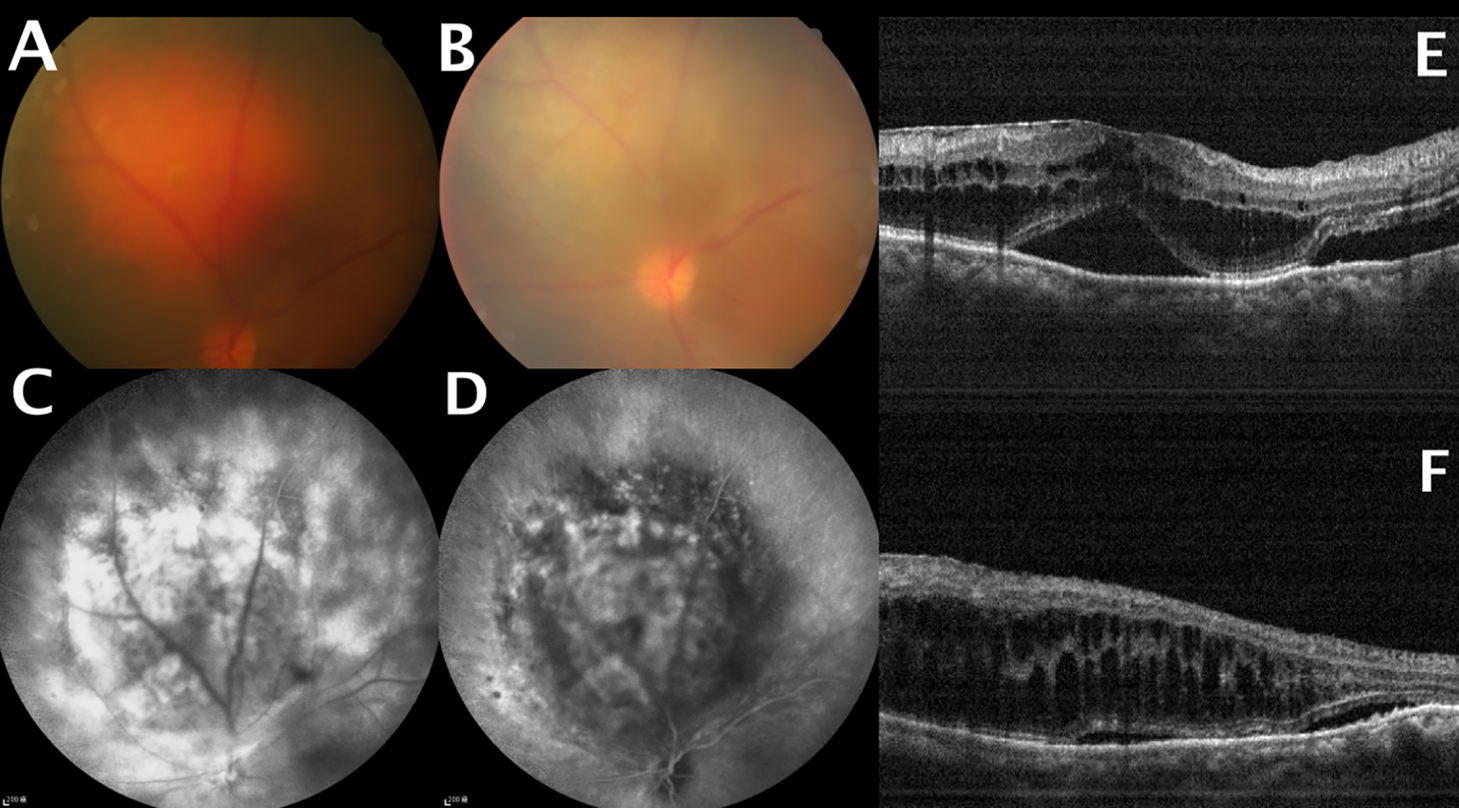
(A) Pretreatment fundus photograph showing a choroidal hemangioma touching the optic disc. (B) Fundus photograph revealed partial tumor regression after five photodynamic therapy (PDT) sessions. (C) Pretreatment fluorescein angiography demonstrated tumor hyperfluorescence. (D) Washout effect on indocyanine angiography. (E) Pretreatment optical coherence tomography (OCT) showing subfoveal fluid accumulation and cystoid macular edema. (F) OCT demonstrating persistent cystoid macular edema and subfoveal fluid accumulation at the last follow-up visit.

Seventeen patients (85%) received one PDT session, whereas three patients (15%) had underwent at least two PDT sessions (patient 1: two sessions; patient 11: three sessions; patient 6: five sessions). Nine patients (45%) in this study received PDT only. Six patients (30%) had been treated with laser photocoagulation in other hospitals before receiving PDT in our center; one patient had a history of previous laser photocoagulation treatment combined with IVI of bevacizumab. In total, seven (35%) patients received IVI of anti-VEGF agents. Among them, six received IVIs of anti-VEGF with PDT as a combined therapy at CGMH and one patient received an IVI of bevacizumab in another institution before being referred to CGMH for PDT. Five patients received a single injection of bevacizumab, one patient (patient 13) was treated with a single injection of ranibizumab, whereas the remaining patient (patient 6) had received five IVIs of bevacizumab and one IVI of ranibizumab for persistent CME and SRF **(Fig 1)**. Patient 11 was treated with external beam radiation (total dose: 1800 cGy) after the failure of three PDT sessions in combination with IVIs of bevacizumab. All patients were followed up for at least 12 months, with the mean follow-up period being 22 months (range: 12−120 months).

### Primary outcome

The decimal BCVA before PDT ranged between 0.02 and 0.5, with a mean of 0.06 ± 0.12 (20/400). Thirteen patients (65%) exhibited a baseline BCVA ≤ 0.10 (20/200).

**Fig 2:**
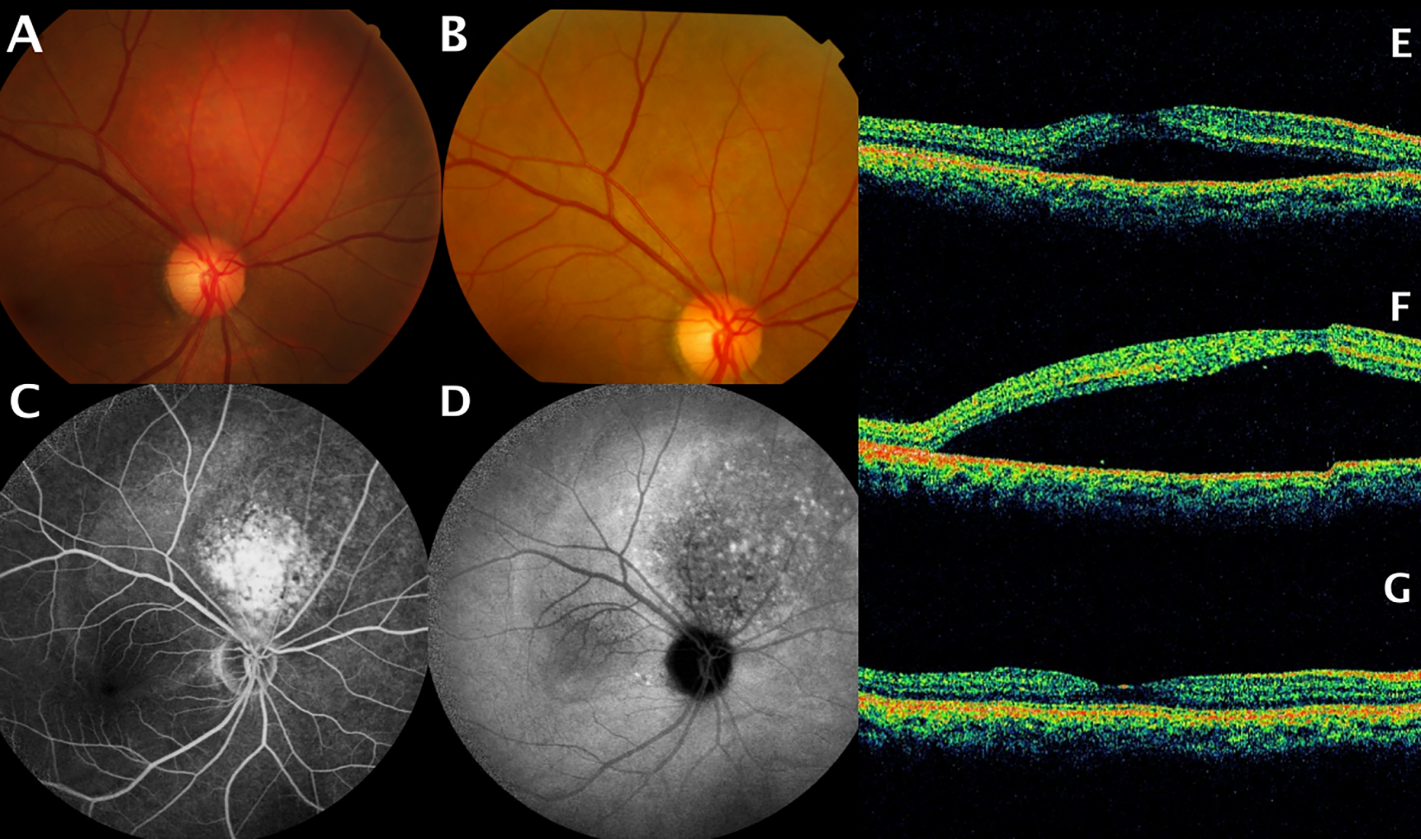
In patient 4, (A) Pretreatment fundus photograph showing a juxtapapillary choroidal hemangioma. (B) Fundus photograph revealed tumor regression after a single photodynamic therapy (PDT) session. (C) Pretreatment fluorescein angiography revealing hyperfluorescence of the tumor and subretinal fluid (SRF) accumulation at the posterior pole. (D) Washout effect on indocyanine angiography. (E) Pretreatment optical coherence tomography (OCT) revealing SRF accumulation. (F) OCT demonstrating a transient CME, macular edema one week after PDT. (G) Complete resolution of SRF accumulation 1 month after PDT.

Overall, the logMAR visual acuity improved significantly (*P* = 0.002) from baseline (median 1.11, range: 0.3–1.70) to posttreatment (median 0.52, range: 0–1.52). The primary outcome measure (i.e. visual improvement as defined in the “Methods”) was achieved by ten patients (50.0%). Among them, nine had received a single PDT session, whereas one (patient 1) had received a second PDT session two years later because of SRF accumulation recurrence. One patient (patient 20) who achieved the primary outcome measure had previously received a single IVI of bevacizumab at another institute before receiving PDT at CGMH; whereas three patients (patients #16, #19, #20) had received focal laser photocoagulation at other institutes before receiving PDT at our institute. Among the 10 patients who achieved visual improvement, 3 patients (patients #4, #9, #19) exhibited a transient increase in SRF accumulation after PDT, which was accompanied by a decreased in BCVA score **(Fig 2)**. Specifically, BCVA decreased from 0.3 (20/60) to 0.1 (20/200), from 0.4 (20/50) to 0.3 (20/60), and from 0.2(20/100) to 0.1 (20/200), respectively; however, the observed deterioration was transient, with subsequent visual improvement was observed in all patients within 1 month [final decimal BCVA of 0.9 (20/25), 0.7 (20/30), and 0.4 (20/80), respectively. The other ten patients exhibited stable post-PDT BCVA scores.

### Secondary outcomes

The median tumor thickness significantly (*P* < 0.001) decreased from a baseline value of 4.0 mm (range: 2.5−7.2 mm) to a post-PDT value of 3.0 mm (range, 0.9−6.5 mm). Similarly, we observed a decrease in median FCT from a pretreatment value of 492 μm (range: 300−852 μm) to 262.5 μm (range: 174–982 μm) after PDT (*P* = 0.019). In patients who received PDT alone, the median tumor thickness values were 3.6mm (range: 2.5–6.0 mm) and 2.0 mm (range: 0.9–5.0 mm) before and after treatment, respectively. In patients who had received additional treatments, the median tumor thickness values were 4.0 mm (range: 2.5–7.2 mm) and 3.6 mm (range: 1.5–6.5 mm) before and after treatment, respectively.There is no significant difference in pretreatment tumor thickness between combined treatment group and monotherapy PDT treatment group (*P* = 0.55). However, the decrease in tumor thickness differed significantly between two groups, namely PDT alone and PDT with additional treatments (*P* = 0.036). The median FCT values before and after treatment with PDT only (n = 9) were 487 μm (range: 300–852 μm) and 233 μm (range: 174–309 μm), respectively. The median FCT values before and after treatments in the group with additional treatments (n = 11) were 497 μm (range: 300–837 μm) and 340 μm (range: 212–982 μm), respectively. The decrease in FCT values differed significantly between two groups (*P* = 0.014). In total, 3 patients (patients #5, #6, #7) had persistent CME. No systemic complications occured during the follow-up period.

### Predictors of primary outcome (BCVA improvement)

The results of bivariate logistic regression analysis are presented in **Table 2**. We identified the following four factors as being significantly associated with the primary outcome measure (BCVA improvement): (1) young age (< 50 years old; OR, 1.176; 95% CI, 1.023−1.352; *P* = 0.023], (2) a pre-PDT BCVA ≥ 20/200 (OR, 16.00; 95% CI, 1.788−143.150; *P* = 0.013), (3) a reduced post-PDT tumor thickness (OR, 7.052; 95% CI, 1.455−34.172; *P* = 0.015), and (4) exudative retinal detachment showing complete resolution within one month after PDT is a factor associated with favorable outcome, (OR, 36.00; 95% CI, 2.721−476.276; *P* = 0.007). In our study, these factors were associated with a favorable outcome. Notably, the additional treatments (including IVI of anti-VEGF) did not exhibit a positive effect on the outcomes **(Table 2)**.

**Table 2:** Univariate logistic regression analysis of factors associated with visual improvement^a^ in patients with symptomatic circumscribed choroidal hemangioma and serous macular detachment.

Factors	OR	95% CI	*P*
Sex (Male vs Female)	1.000	0.112–8.947	1.000
Age (>50 years vs ≤ 50 years)	9.333	1.193–72.991	0.033
Symptom duration	1.041	0.948–1.142	0.399
Presented BCVA ≥ 20/200	16.000	1.788–143.150	0.013
Tumor location (JP VS MA)	0.444	0.074–2.660	0.374
Minimal dimension of initial tumor basal diameter	1.335	0.808–2.205	0.260
Maximal dimension of initial tumor basal diameter	1.172	0.814–1.688	0.394
Initial tumor thickness	2.149	0.907–5.090	0.082
Initial foveal center thickness	1.000	0.995–1.005	0.992
Transient exudative retinal detachment	36.000	2.721–476.276	0.007
Final tumor thickness	7.052	1.455–34.172	0.015
Final foveal center thickness	1.011	0.996–1.026	0.154
Sessions of PDT	2.250	0.170–29.767	0.538
PDT combined with intravitreal drug injection	4.000	0.550–29.096	0.171
PDT and failed focal retinal photocoagulation	1.000	0.148–6.772	1.000

^a^Defined as an improvement of at least two lines in BCVA after treatment and a final BCVA ≥ 20/60.

An OR > 1 indicates an increased likelihood of having visual improvement.

Abbreviations: OR = odds ratio; CI = confidence interval; BCVA = best corrected visual acuity; PDT = photodynamic therapy.

## Discussion

Consistent with several previous studies [5,6,7,8,9,10,11,12], the results of our case series confirm that symptomatic CCHs respond generally well to PDT, because 50% of patients exhibited a significant visual improvement. In particular, we identified young age (<50 years) and a pre-PDT BCVA ≥ 20/200 as pretreatment variables significantly associated with an improved post-treatment visual acuity. Lower rates of chorioretinal atrophy or degenerative changes of the posterior pole may contribute to more favorable visual outcomes in younger patients [20,21]. In addition, exudative retinal detachment showing complete resolution within 1 month after PDT and a reduced post-PDT tumor thickness were identified as post-treatment variables associated with an improved BCVA. Although PDT is currently considered as the treatment of choice for symptomatic submacular and peripapillary CCHs [5,6,7,8,9,10,11,12], some patients cannot afford its high cost. In this context, the selection of ideal candidates who are most likely to benefit from this approach is necessary. The present study was primarily designed to address this knowledge gap. A few recent studies have investigated the effectiveness of IVI of anti-VEGF in treating symptomatic CCHs [13,14,15,16].^.^We also performed an exploratory analyzed whether the combination of PDT with other therapies is significantly associated with more positive outcomes than is PDT alone. Albeit preliminary in nature, our data did not indicate a significant added value of other treatments in combination with PDT when compared with PDT alone. The positive effects of PDT as monotherapy were evident despite the long delay between symptom onset and treatment observed in the current series (median duration of symptoms before presentation: 2 months). The potential correlation between symptom duration before PDT and visual outcomes remains controversial. Some authors have suggested that longer symptom duration may be associated with a poor final visual acuity (≤ 20/200) [1,2], whereas other reports have not [8,9,10,11,12]. In our study, binary logistic regression analysis did not identify symptom duration as a significant predictor of visual outcomes. In contrast, our results indicated that patients aged <50 years with a pre-PDT BCVA score of> 20/200 are ideal candidates for PDT. Notably, most of our patients required only a single PDT session to achieve an improved visual acuity. The factor ‘multiple PDT sessions’ is inversely related to favorable visual outcomes.

In our series, changes in tumor anatomical parameters served as secondary outcome measures. Notably, the mean baseline tumor size of patients in our study was observed to be relatively larger than that of patients in previous case series [6,7,11,13,14,15,16]. Before treatment, there were only two patients had a tumor thickness of < 3 mm. The tumor thickness and FCT decreased after PDT, without significant differences between patients who had received PDT alone and those who had received PDT combined with other therapies. Notably, a reduced in post-PDT tumor thickness was associated with favorable visual outcomes. In total, local complications occured in eight patients and were generally associated with poor visual outcomes. The question of whether such complications are related to PDT *per se* or are part of the natural course of CCHs remains unanswered [2,3,18]. Notably, three patients exhibited a transient increased SRF accumulation after treatment. This phenomenon has been previously reported in patients treated with PDT for intraocular tumors and has been related to post-PDT visual improvements [22,23]. Increased SRF accumulation may be explained by the occurrence of vascular thrombosis in the rich vascular bed typical of CCHs. However, IVIs of anti-VEGF was not beneficial for reducing CME in our series.

Our study has several limitations; hence, our results should be interpreted in the appropriate context. First, case series of symptomatic CCH are invariably limited by small sample sizes. Second, the addition of other treatments to PDT was not randomized; consequently, our results indicating that the use of additional treatments was not associated with additional improvements in visual acuity (*versus* PDT alone) should be interpreted with caution. The challenges in conducting a head-to-head comparative randomized clinical trial to provide sufficient evidence in rare diseases are enormous. Furthermore, the study population was non-representativeness of the actual population because our study was set at a tertiary referral center. Notably, our patients mostly had advanced CCHs, histories of previously failed treatments, long delays between symptoms and treatment, and poor pretreatment visual acuity. In particular, most of the CCHs in our series were observed to be larger than those described in previous reports [5,6,7,11,13,14,15,16]. Large CCHs may exhibit relatively mature, stabilized blood vessels that are less likely to respond to IVIs of anti-VEGF on other causes, such as age related macular degenerations, or diabetic macular edema [24].

In conclusion, the results of our case series indicate that PDT is safe and effective in the treatment of CCHs. PDT is still effective on these symptomatic previously treated tumors. Similar results were also reported by Jurkies *et al* [12] and Singh *et al*. [23]. Patients aged < 50 years and with a pretreatment visual acuity better than 20/200 are most likely to benefit from PDT. Patients who do not meet these criteria should be informed of the risks of suboptimal visual outcomes before undergoing PDT. We also demonstrated that exudative retinal detachment showing complete resolution within 1 month and reduced post-PDT tumor thickness are significant predictors of favorable post-treatment outcomes. The combination of PDT with other therapies (including IVI of anti-VEGF) and multiple PDT sessions cannot currently be recommended.
